# Smart city perspectives in post-pandemic governance: Externalities reduction policy

**DOI:** 10.12688/f1000research.123195.1

**Published:** 2022-09-12

**Authors:** Olga Vladimirovna Kalinina, Sergey Evgenievich Barykin, Sergey Mikhailovich Sergeev, Galina NikolaevnaSemenova, Alina Fatkullina, Alexey Mikhaylov, Elena De La Poza Plaza

**Affiliations:** 1 Graduate School of Industrial Management, Peter the Great St. Petersburg Polytechnic University, St. Petersburg, Russian Federation; 2 Graduate School of Service and Trade, Peter the Great St. Petersburg Polytechnic University, St. Petersburg, Russian Federation; 3 Department of Accounting and Taxation, Plekhanov Russian University of Economics, Moscow, Russian Federation; 4 Department of Engineering Graphics and Computer Modeling, Moscow State University of Civil Engineering, Moscow, Russian Federation; 5 Moscow Architectural Institute, Moscow, 107031, Russian Federation; 6 Department of Banking and Financial Markets, Financial University under the Government of the Russian Federation, Moscow, Russian Federation; 7 Center for Economic Engineering (INECO), UniversitatPolitècnica de València, Valencia, Spain

**Keywords:** Digital Interactions, Smart Cities, Sustainable Development, Digital Platforms

## Abstract

**Background:** The ongoing COVID-19 quarantine restrictions have caused multiple sharp decreases in activities associated with the movement of large masses of people. The economies of regions and cities that are critically dependent on tourist flows related to various segments have suffered. This research aims to provide an economic-mathematical model of smart cities externalities’ impact from the point of view of achieving social and environmental goals

**Methods:** The objective of this study was to develop an algorithm for supporting decision-makers. Methods of mathematical modeling, statistical processing of data received in real-time, as well as methods for finding solutions by expansion into dynamic series are used, and the theory of mathematical games is applied. The theoretical mathematical model presented considers the statistical processing of data provided in real time referring to the performance indicators of megacities.

**Results:** The activities of administrations and governments aimed at maintaining stability over the past two years have been aimed at reducing the negative impact of the pandemic. The prospect of returning to normal conditions is complicated by a number of factors. The proposed approach allows the development of the fundamental basis for making administrative decisions within individual megapolises and in environmental policy on a territory of any scale. The developed mathematical model is abstract by definition and is applied by taking into account specific tasks and criteria. Since the tasks of the administration differ depending on the region and country, the choice of criteria is set individually.

**Conclusions:** During the period of isolation, the volume of services in the Hotel - Restaurant- Catering/Café (HORECA) segment has decreased, and personnel has also been lost. The reduced pressure on public infrastructure and the departure of migrants means that, in the long term, this work cannot be restored within a short period of time.

## Introduction

Since the late 1990s, thanks to innovations in several decisions and globalization, running a business and the public’s way of life have changed significantly. ^1^ New opportunities for fulfilling one’s potential become available to active people. The development of telecommunications systems allows you to organize virtual development teams, while distance learning also reduces the need to be in specific places. The mobility of the labor force became possible due to the elimination of border and customs barriers, and the transport infrastructure was additionally developed, especially in Europe. Therefore, people, especially the younger generation, have become less attached to a settled way of life and choose dynamic movement between locations. At the same time, alpha cities and megacities, which provide maximum opportunities for self-realization, become centers of attraction. The movement of mobility of the labor force within Europe and between other states has led to an increase in the dynamics of the structure of employment of the population. International transport services of all kinds have become available over the world (the proposed mathematical model is equally acceptable for any regions of the world.).

The authors consider the coronavirus crisis as a test of whether smart city technology may mitigate the negative impacts. The COVID-19 pandemic was a black swan event that disrupted the established chains of interaction and completely reshaped the economic landscape of most regions and cities of the world, which are centers of attraction for human flows. ^2^ The researchers could define the cities with a preparedness resiliency plan as ‘smarter’ using the Internet-of-Things (IoT) sensors, cameras etc. ^1^ Digital transformation allows many businesses to develop seamless working-from-home experiences. The degree of equipment with technical means for analyzing current information about the state of urban infrastructure, its processing for different cities and regions is different. This often depends on the budget of the administration and on legislative restrictions. The most advanced control systems today are in Southeast Asia, such as China, but during the COVID-19 pandemic, telecommunications and data processing systems have been rapidly developing throughout Europe. The importance of IoT systems and machine-to-machine interaction has also increased manifold. First of all, this is the automatic collection of data on the migration of people, traffic on the roads, the expenditure of all types of resources, and the general situation in cities. In addition, BigData technologies based on predictive algorithms make it possible to make decisions with an effect extended over time. The anti-pandemic measures have accelerated broadband consumption growth by over 60%. ^3^

The problem of post-pandemic governance could be described from the point of view of organizing business interaction within the consumer value chain. The idea of forming a consumer value chain was initially proposed by M. Porter in 1985. ^4^ This considers the term ‘echelon’ which relates to the theoretical approaches of modeling multi-echelon supply chain design ^5^ and social sustainability assessment of upstream (a set of connected firms that involve raw material extraction and transformation), midstream (production and assembly), and downstream (sales and services) echelons of supply chains. ^6^^–^^9^ Some researchers explore solid waste management systems from the logistics and supply chains theory ^10^ and multi-echelon supply chains under stochastic and fuzzy environments. ^11^

The upcoming removal of restrictive quarantine measures will have a shocking impact on all services and will require the administrations of regions and cities to make informed and calculated decisions. ^12^ The lack of theoretical propositions regarding sustainable development goals supporting the quality of life in the post-pandemic period refers to the research gap. The authors are attempting to investigate environmental and social goals from the point of view of administrative measures to conquer the negative consequences of coronavirus disease, considering an ecological policy. The authors propose exploring digital transformation in smart cities as a tool for sustainable development, making the logical bridge between the theoretical framework of sustainability concept and common property resources as an environmental asset in a digital era.

This article aims to provide an economic-mathematical model of smart cities externalities’ impact from the point of view of achieving social and environmental goals. The result is a set of formalisms ^13^^,^^14^ that allow the application of scientific methods to find optimal solutions. ^15^^,^^16^ The proposed approach allows the development of the fundamental basis for making administrative decisions within individual megapolises and in environmental policy fora territory of any scale.

## Methods

### Study design

The authors propose the extension of the theoretical construction of the process of interaction between the business echelons within value chains. ^17^ The considered algorithm has been validated in the works. ^17^^,^^18^

The researchers consider the definition of the business echelons as various stages of the consumer value chain regarding the complete promotion cycle of any product taking into account the features of digital ecosystems. ^19^ The digital transformation of both logistics networks and marketing channels is a tool for implementing the smart city perspectives in post-pandemic governance. ^20^ The basis for this study was the theoretical research on the workload of megapolises. We suggest considering Alpha cities as centers of the following group of activities:

‐ business events, meetings, incentives, conferences, and exhibitions corporate segment;
‐ the flow of people attending landmark social and sporting events, and social life phenomena;
‐ sightseeing, places of interest (POI) of cities of the level from Alpha to Alpha ++ by the Globalization and World Cities Research network (GaWC) ^21^ rating;
‐ medical services, modern hospital centers;
‐ social mobility of workers in megapolises;
‐ student mobility (a characteristic feature of most megapolises). ^22^

### Data collection

The dependency regarding the mentioned factors post-pandemic, could be considered stochastic based on quantitative data, reflecting demand's seasonality with an annual frequency. The authors group this set of indicators according to the following main features: determined by the business environment; restrictions of a natural nature; factors regulated by the authorities; restrictions dictated by cultural, religious and national characteristics. Through mathematical analysis, ^23^^,^^24^ we decomposed this dependency into a Fourier series to deconstruct its complex form into several harmonic components (each of them being a periodic function). The authors considered the seasonality function of the loading resource. The pronounced seasonality is due to the multiple growth of tourism during summer holidays, vacations of students several times a year, periodic international events such as sports competitions, cultural festivals, public holidays, Christmas holidays, etc. The use of the Fourier method makes it possible to obtain an answer to the main question of the study. In this work, it is the analytical solution that serves as the basis for the development of predictive software products. Since a lot of time passes between the adoption of decisions of all levels of government in megacities and their implementation, the possibilities of mathematical modeling allow city administrations to take the necessary measures in advance to prepare for a sharp change in the conditions for the functioning of all city services. The exit from the pandemic has led to a dramatic change in living conditions. It also imposes extraordinary requirements for adaptation for all levels of life organization of any territories and any specifics.

### Data analysis

This article obtained an analytical solution in quadratures, scalable to each system. Using the superposition of the obtained solutions with weights corresponding to the initial decomposition, it became possible to get an integrated dependency. ^25^ The proposed approach allowed formalizing the resources of the megapolis in the form of a dynamic task. The researchers achieved results in a group of solutions based on the dependence ^26^ of the environmental costs. In our study, groups of environmental factors are identified, such as restrictions on utilities in terms of waste, sewage, drinking water supply, throughput of highways, taking into account air pollution by road and other transport, the limiting capabilities of medical institutions and the permissible anti-epidemic level of concentration of people. In addition, we additionally take into account restrictions on the supply of the territory with all types of material life support and the limiting capabilities of public order services. The authors recommended the administrative regulation for decision-making, improving the quality of life in megapolis based on the developed theoretical approach considering the digital transformation as a tool for sustainable development. We have proposed a mathematical model, brought to the result of the solution. The specific application of the developed formalisms depends on the tasks and priorities set and may vary for different cities and regions. The authors used the Fourier method aiming to obtain an answer to the main question of the study to test the formalisms presented in the results section. In this work, the researchers are attempting to develop the analytical solution that serves as the basis for the development of predictive software products. The methods used in the study belong to the category of mathematical modeling and big data processing technologies. Authors paid special attention should to the use of decomposition of complex dependencies into harmonic components.

## Results

The construction of a mathematical model is necessary to select optimal solutions. The new industry 4.0 technologies (e.g., cyber physics systems, big data, IoT, etc.) allow for extending the field of financial logistics implementation based on the digital transformation of logistics ^18^^,^^27^ and digital twins. ^26^ We used the technology of developing a digital twin, which allowed us to apply the methods developed in mathematics to find the optimal result. The authors used the concept of digital twin being developed in previous works regarding digital logistics. ^26^^,^^28^ This research aims to develop digital twins of the considered process. The authors introduce several notations for the arguments used. ^29^

The dependence of the load on time t is $\Lambda \left(t\right)$ . Since $\Lambda \left(t\right)$ changes unevenly throughout the year, reflecting the load acting on the resources available in the region or the megapolis, we will represent its decomposition as a set $\lambda_{i}\left(t\right)$ (where i=1,2,… ). The authors introduce a periodicity T characteristic of the load on resources. T is equal to the annual cycle. The researchers define a set $\nu_{i}\left(t\right)$ for the values of the probabilities of the use of the common resources of the territory. Next, we introduce $E_{k}\left(t\right)$ functions that define a set of system states together with a set of their probabilities:

$$p_{i}\left(t\right)=\varphi_{i}\left(t\right)+\alpha_{i}\left(t\right).$$

In this expression, $\varphi_{i}\left(t\right)$ is the T/2πi periodic, and the term $\alpha_{i}\left(t\right)$ satisfies the condition $\lim_{t\to \infty }\alpha_{i}\left(t\right)=0$ , and ∀i . In this interpretation, $\varphi_{i}\left(t\right)$ reflects the stationary probability $p_{i}\left(t\right)$ . Next, we can consider the sequence:

$$p_{0i}^{\prime }\left(t\right)=-\lambda_{i}\left(t\right)p_{0i}\left(t\right)+\nu_{i}\left(t\right)p_{1i}\left(t\right),\;p_{1i}^{\prime }\left(t\right)=-\nu_{i}\left(t\right)p_{1i}\left(t\right)+\lambda_{i}\left(t\right)p_{0i}\left(t\right)$$

together with the complete group condition:

$$\sum_{i}p_{i}\left(t\right)=1.$$

After carrying out an algebraic transformation of the form:

$$p_{0i}^{\prime }\left(t\right)=-\left[\lambda_{i}\left(t\right)+\nu_{i}\left(t\right)\right]p_{0i}\left(t\right)+\nu_{i}\left(t\right)$$

at zero conditions

$$p_{0i}\left(0\right)=\alpha_{i}\leq 1,\;p_{1i}\left(0\right)=1-\alpha_{i}\leq 1,$$

it becomes possible to obtain the desired system already in quadratures in the form:

$$p_{0i}\left(t\right)=e^{-\int_{0}^{t}\left[\lambda_{i}\left(x\right)+\nu_{i}\left(x\right)\right]\mathrm{dx}}\left[\alpha_{i}+\int_{0}^{t}\nu_{i}\left(x\right)e^{-\int_{0}^{x}\left[\lambda_{i}\left(z\right)+\nu_{i}\left(z\right)\right]\mathrm{dz}}\mathrm{dx}\right].$$

The authors denote $\varphi_{i}\left(t\right)=\int_{0}^{t}\left[\lambda_{i}\left(x\right)+\nu_{i}\left(x\right)\right]\mathrm{dx}$ , as well as k , which satisfies the inequality: $\mathrm{kT}\leq t<\left(k+1\right)T$ . The researchers suggest an expression for the calculation of $\varphi_{i}\left(t\right)$ :

$$\varphi_{i}\left(t\right)={\mathrm{ka}}_{i}+\int_{0}^{\tau }\left[\lambda_{i}\left(z\right)+\nu_{i}\left(z\right)\right]\mathrm{dz},$$

Here, it is assumed that: $a_{i}=\int_{0}^{T}\left[\lambda_{i}\left(x\right)+\nu_{i}\left(x\right)\right]\mathrm{dx}$ , τ=t−kT .

Similarly, the authors express: $\int_{0}^{t}\nu_{i}\left(x\right)e^{\varphi_{i}\left(x\right)}\mathrm{dx}=\sum_{l=1}^{k}\int_{\left(l-1\right)T}^{\mathrm{lT}}\nu_{i}\left(x\right)e^{\varphi_{i}\left(x\right)}\mathrm{dx}+\int_{\mathrm{kT}}^{t}\nu_{i}\left(x\right)e^{\varphi_{i}\left(x\right)}\mathrm{dx}$ , and this gives us $p_{0i}\left(t\right)=e^{-\varphi_{i}\left(\tau \right)}\left[\frac{b_{i}}{e^{a}-1}+\int_{0}^{\tau }\nu_{i}\left(x\right)e^{\varphi_{i}\left(x\right)}\mathrm{dx}\right]+e^{-\mathrm{ka}-\varphi_{i}\left(\tau \right)}\left[\alpha_{i}-\frac{b_{i}}{e^{a}-1}\right]$ ,

In this case, the designation for the parameter is introduced: $b_{i}=\int_{0}^{T}\nu_{i}\left(x\right)e^{\varphi_{i}\left(x\right)}\mathrm{dx}$ .

The authors search for the optimal solution on the obtained basis ^30^ using the common approach of digital twin concept. ^13^^,^^31^^,^^32^ To do this, we formulate the problem in a general form. The authors then define the number of users of the resources as n . In this case, the load $l_{i}$ , where i=1…n , reflects the of each user’s externalities for the vector: $\bar{L}=\left(l_{1},l_{2},\ldots ,l_{n}\right)$ .

The total load $L^{*}=\sum_{i=1}^{n}l_{i}$ .

The researchers introduce the weighted average ^14^^,^^30^^,^^33^^–^^35^ variable costs $U_{i}$ and the dependence $g_{i}\left(l_{i}\right)$ of the revenue of each user. Due to the limited capacity of the shared resource, starting from $\bar{L}>{\bar{L}}_{R}$ , the profitability drops. This corresponds to the fulfillment of the condition $g_{i}^{\prime }\left(l_{i}\right)<0$ . Moreover, due to the negative impact of the externalities of the specified n users, the second derivative is subject to the condition $g_{i}^{\prime \prime }\left(l_{i}\right)<0$ .

Then, the profit of the i− th user is $R_{i}=l_{i}g\left(l_{1}+l_{2}+\ldots +l_{n}\right)-U_{i}l_{i}=l_{i}g_{i}\left(L\right)-U_{i}l_{i}$ . Based on the Nash equilibrium principle, ^36^ there is a value $l_{i}^{*}$ for i=1…n , at which $R_{i}$ reaches a maximum for any components $\bar{L}$ : ${\bar{L}}_{i}^{*}\left(l_{1}^{*},l_{2}^{*},\ldots ,l_{i-1}^{*},l_{i+1}^{*},\ldots ,l_{n}^{*}\right)$ . We find the extremum points from the conditions (for the calculation): $\frac{\partial R_{i}}{\partial l_{i}}=0$ , at i=1…n . Denoting the sum $l_{-i}^{*}=\sum_{k\neq i}l_{k}^{*}$ , the authors obtain $g\left(l_{i}+l_{-i}^{*}\right)+l_{i}g^{\prime }\left(l_{i}+l_{-1}^{*}\right)-U=0$ for i=1…n , which gives the desired value of $L^{*}=n\frac{U-g\left(L^{*}\right)}{g^{\prime }\left(L^{*}\right)}$ . Consequently, the extremum is reached at $L_{0}=\frac{U-g\left(L_{0}\right)}{g^{\prime }\left(L_{0}\right)}$ . Since the condition n>1 is satisfied, it follows that $L^{*}>L_{0}$ . This proves the obtained result.

The mathematical model formulated in the article provides an opportunity for developing several software applications (however the authors are attempting to develop the common theoretical approach). Since the calculations are scalable, it is possible to cover most of the critical areas of economic activity that affect the quality of life, the environment, and the economic performance of a given territory. First of all, the authors were guided by publicly available specifications V182 ( https://www.bsigroup.com/en-GB/smart-cities/Smart-Cities-Standards-and-Publication/PAS-182-smart-cities-data-concept-model/ accessed on 27 April, 2022). In this case, we consider the result as an algorithm acceptable for embedding in the smart city digital platform. The proposed approach allows the development of the fundamental basis for making administrative decisions within individual megapolises and in environmental policy for the territory of any scale. The developed mathematical model is abstract by definition and is applied taking into account specific tasks and criteria. The theoretical recommendations are expected to be implemented for designing a customer value chain and digital logistics network in smart cities and smart territories. The governing measures consider planning optimal organization of suppliers in a digital logistics network, taking into account the interests of stakeholders and the priorities of the sustainability concept. The developed method will add value to the field of planning smart cities and smart territories based on the model including the economic interests of customers, profit indicators, the delivery time of goods, and reliability of supplies. The usage of this method allows customer value creation by developing a customer-oriented approach and achieving public consensus on an integrated network structure. The expected value of peer review could provide the best available path for designing smart cities’ logistics networks to gain social, environmental, and governing goals of the sustainable development concept.

## Discussion

The authors suggest discussing the wide area of issues regarding the smart city concept based on digital interaction within a constantly evolving sustainable environment. Due to the publicly available specifications, the decision model has grown significantly. Big data technologies, high-speed mobile means of information transmission, and urban management algorithms have become the norm. However, predictive methods are required for black swan events such as the COVID-19 pandemic. ^37^^–^^39^ The evolution of smart cities could be considered in the context of the discussion of the developed algorithm compared to existing research. The authors are attempting to investigate the complex task of the sustainable development in post-pandemic situation. The existing approaches consider the COVID-19 pandemic as a continuous crisis having a negative impact on the sustainable development, but do not take into account the difficulties caused by post-pandemic crisis. The researchers aimed to develop an approach considering the digital transformation as a tool for achieving Environmental, Social, Governmental (ESG) goals with supporting measures as a common resource access policy.

The authors believe that decision-makers need predictive methods for the post-COVID-19 transition period. Undoubtedly, removing restrictions will entail serious negative consequences for the economy, ecology, and municipal infrastructure of megapolises. It is necessary to calculate the optimal mode of interaction between the administration, government, and experts in commerce and the service sector that determines the lives of the population and arriving people. The developed model solves the most challenging task of organizing an increase in the volume of services concentrated in a limited location while minimizing negative externalities. The framework of the developed approach is limited by mathematical assumptions and methods of game theory. The authors propose a topic for future research related to digital transformation as a tool for achieving sustainable development goals.

Exploring methods and models of digital logistics relying on different conditions of smart cities’ sustainable development could be a topic for future research in a coupe with several points of view in works. ^13^^,^^40^^–^^42^

## Conclusions

The authors considered a systematic approach comprising the sustainable development concept and smart cities’ evolution based on digital technologies. This article obtained an analytical solution in quadratures, using the superposition of the obtained solutions with weights corresponding to the initial decomposition. The proposed approach allowed formalizing the resources of the megapolis in the form of a dynamic task. The researchers achieved results in a group of solutions based on the dependence of the environmental costs. The authors recommend the administrative regulation for decision-making, improving the quality of life in megapolis considering the digital transformation as a tool for achieving ESG goals.

Currently, the administrative regulation measures for supporting sustainable development in megapolises use heuristic methods in their current activities. The severe disruption caused by the pandemic has shown the unsustainability of the current management practices. No dynamic analysis and search for optimal solutions are carried out in the decision-making process. Such difficulties are primarily due to the complexity of considering many factors and the lack of scientifically based theoretical models that consider economic indicators. However, the upcoming release from the restrictive measures of the quarantine regime can lead to much greater shocks. The developed methodology makes it possible to calculate the balanced benefit for all participants in the activity, taking into account externalities and choosing alternative options.

## Data availability

No data are associated with this article.

## References

[1] Prataviera E Vivian J Lombardo G : Evaluation of the impact of input uncertainty on urban building energy simulations using uncertainty and sensitivity analysis. *Appl. Energy.* 2022 Apr;311(November 2021):118691. 10.1016/j.apenergy.2022.118691

[2] Aust I Matthews B Muller-Camen M : Common Good HRM: A paradigm shift in Sustainable HRM? *Hum. Resour. Manag. Rev.* 2020 Sep;30(3):100705. 10.1016/j.hrmr.2019.100705 Reference Source

[3] MSCI: Annual Report. 2020. Reference Source

[4] Porter ME : *Competitive Advantage: Creating and Sustaining Superior Performance.* New York: The Free Press, Macmilan;1985;557p.

[5] Yue D You F : Game-theoretic modeling and optimization of multi-echelon supply chain design and operation under Stackelberg game and market equilibrium. *Comput. Chem. Eng.* 2014;71:347–361. 10.1016/j.compchemeng.2014.08.010

[6] Popovic T Barbosa-Póvoa A Kraslawski A : Quantitative indicators for social sustainability assessment of supply chains. *J. Clean. Prod.* 2018;180:748–768. 10.1016/j.jclepro.2018.01.142

[7] Ullah M Asghar I Zahid M : Ramification of remanufacturing in a sustainable three-echelon closed-loop supply chain management for returnable products. *J. Clean. Prod.* 2021;290:125609. 10.1016/j.jclepro.2020.125609

[8] Sarkar B Guchhait R Sarkar M : Impact of safety factors and setup time reduction in a two-echelon supply chain management. *Robot. Comput. Integr. Manuf.* May 2017;55(55):250–258. 10.1016/j.rcim.2018.05.001

[9] Mitra S : Inventory management in a two-echelon closed-loop supply chain with correlated demands and returns. *Comput. Ind. Eng.* 2012;62(4):870–879. 10.1016/j.cie.2011.12.008

[10] Zhang Y Huang GH He L : A multi-echelon supply chain model for municipal solid waste management system. *Waste Manag.* 2014;34:553–561. 10.1016/j.wasman.2013.10.002 24268473

[11] Gumus AT Guneri AF : A multi-echelon inventory management framework for stochastic and fuzzy supply chains. *Expert Syst. Appl.* 2009;36(3 PART 1):5565–5575. 10.1016/j.eswa.2008.06.082

[12] Popkova E DeLo P Sergi BS : Corporate Social Responsibility Amid Social Distancing During the COVID-19 Crisis: BRICS vs. OECD Countries. *Res. Int. Bus. Financ.* 2021 Jan;55:101315. 10.1016/j.ribaf.2020.101315 Reference Source 34173410 PMC7434437

[13] Barykin SE Borisoglebskaya LN Provotorov VV : Sustainability of Management Decisions in a Digital Logistics Network. *Sustainability.* 2021 Aug 18;13(16):9289. 10.3390/su13169289 Reference Source

[14] Zhabko AP Shindyapin AI Provotorov VV : Stability of weak solutions of parabolic systems with distributed parameters on the graph. *Vestn. Saint Petersbg. Univ. Appl. Math. Comput. Sci. Control Process.* 2019;15(4):457–471. Reference Source

[15] Provotorov VV Sergeev SM Hoang VN : Countable stability of a weak solution of a parabolic differential-difference system with distributed parameters on the graph. *Vestn. Saint Petersbg. Univ. Appl. Math. Comput. Sci. Control Process.* 2020;16(4):402–414. 10.21638/11701/spbu10.2020.405 Reference Source

[16] Provotorov VV Sergeev SM Nguyen H Van : Point control of a differential-difference system with distributed parameters on the graph. *Vestn. Saint Petersbg. Univ. Appl. Math. Comput. Sci. Control Process.* 2021;17(3):277–286. 10.21638/11701/spbu10.2021.305 Reference Source

[17] Barykin SE Smirnova EA Chzhao D : Digital Echelons and Interfaces within Value Chains: End-to-End Marketing and Logistics Integration. *Sustainability.* 2021 Dec 16;13(24):13929. 10.3390/su132413929 Reference Source

[18] Shmatko A Barykin S Sergeev S : Modeling a Logistics Hub Using the Digital Footprint Method—The Implication for Open Innovation Engineering. *J. Open Innov. Technol. Mark. Complex.* 2021 Feb 7;7(1):59. 10.3390/joitmc7010059 Reference Source

[19] Barykin SY Kapustina IV Kirillova TV : Economics of Digital Ecosystems. *J. Open Innov. Technol. Mark. Complex.* 2020 Oct 22;6(4):124. 10.3390/joitmc6040124 hReference Source

[20] Miroshnyk NV Likhanov AF Grabovska TO : Green infrastructure and relationship with urbanization – Importance and necessity of integrated governance. *Land Use Policy.* 2022 Mar;114(January 2021):105941. 10.1016/j.landusepol.2021.105941

[21] Globalization and World Cities Research Network.accessed on 27 ^th^of April, 2022. Reference Source

[22] e Silva LC Costa APCS : Decision model for allocating human resources in information system projects. *Int. J. Proj. Manag.* 2013;31(1):100–108. 10.1016/j.ijproman.2012.06.008

[23] Provotorov VV Provotorova EN : Optimal control of the linearized Navier— Stokes system in a netlike domain. *Vestn. Saint Petersbg. Univ. Appl. Math. Comput. Sci. Control Process.* 2017;13(4):431–443. 10.21638/11701/spbu10.2017.409 Reference Source

[24] Podvalny SL Podvalny ES Provotorov VV : The Controllability of Parabolic Systems with Delay and Distributed Parameters on the Graph. *Procedia Comput. Sci.* October 2016;103(103):324–330. 10.1016/j.procs.2017.01.115

[25] Golosnoy AS Provotorov VV Sergeev SM : Software engineering math for network applications. *J. Phys. Conf. Ser.* 2019 Dec 1;1399(4):044047. 10.1088/1742-6596/1399/4/044047

[26] Barykin SY Kapustina IV Sergeev SM : Developing the physical distribution digital twin model within the trade network. *Acad. Strateg. Manag. J.* 2021;20(1):1–18. Reference Source

[27] Barykin SY Bochkarev AA Dobronravin E : The place and role of digital twin in supply chain management. *Acad. Strateg. Manag. J.* 2021;20(2):1–19. Reference Source

[28] Kapustina I Bakharev V Barykin S : Digitalization of logistics hubs as a competitive advantage of logistics networks. Kudriavtcev S, Murgul V, editors. *E3S Web Conf.* 2020 Mar 20;157:05009. 10.1051/e3sconf/202015705009

[29] Barykin SY Kapustina IV Sergeev SM : Algorithmic Foundations of Economic and Mathematical Modeling of Network Logistics Processes. *J. Open Innov. Technol. Mark. Complex.* 2020 Dec 13;6(4):189. 10.3390/joitmc6040189 Reference Source

[30] Borisoglebskaya LN Provotorov VV Sergeev SM : Mathematical aspects of optimal control of transference processes in spatial networks. *IOP Conf. Ser. Mater. Sci. Eng.* 2019;537(4):042025. 10.1088/1757-899X/537/4/042025

[31] Bril A Kalinina O Barykin S : The Methodological Features of the Economic Evaluation of Personnel Management Operational Projects. *Commun. Comput. Inf. Sci.* 2020:143–154. 10.1007/978-3-030-60080-8_8

[32] Barykin S Smirnova E Sharapaev P : Global Supply Chains Development on the Basis of Russia and Kazakhstan Economies Digitalization. *Proceedings of the International Conference on Digital Technologies in Logistics and Infrastructure (ICDTLI 2019).* Paris, France: Atlantis Press;2019; p.235–40. Reference Source

[33] Pilipenko OV Provotorova EN Sergeev SM : Automation engineering of adaptive industrial warehouse. *J. Phys. Conf. Ser.* 2019;1399(4):044045. 10.1088/1742-6596/1399/4/044045

[34] Artemov MA Baranovskii ES Zhabko AP : On a 3D model of non-isothermal flows in a pipeline network. *J. Phys. Conf. Ser.* 2019;1203(1):012094. 10.1088/1742-6596/1203/1/012094

[35] Krasnov S Sergeev S Zotova E : Algorithm of optimal management for the efficient use of energy resources. Kalinina O, editor. *E3S Web Conf.* 2019 Aug 9;110:02052. 10.1051/e3sconf/201911002052

[36] De Persis C Grammatico S : Distributed averaging integral Nash equilibrium seeking on networks. *Automatica.* 2019 Dec;110:108548. 10.1016/j.automatica.2019.108548

[37] Santis S : The Demographic and Economic Determinants of Financial Sustainability: An Analysis of Italian Local Governments. *Sustainability.* 2020 Sep 15;12(18):7599. 10.3390/su12187599 Reference Source

[38] Giordano G : A Hybrid Supply Chain. *Plast. Eng.* 2020 May 6;76(5):9–11. 10.1002/peng.20304

[39] DeWit A Shaw R Djalante R : An integrated approach to sustainable development, National Resilience, and COVID-19 responses: The case of Japan. *Int. J. Disaster Risk Reduct.* 2020 Dec;51(August):101808. 10.1016/j.ijdrr.2020.101808 32834979 PMC7425667

[40] Pouri MJ Hilty LM : Conceptualizing the Digital Sharing Economy in the Context of Sustainability. *Sustainability.* 2018 Nov 27;10(12):4453. 10.3390/su10124453 Reference Source

[41] Bevilacqua M Bottani E Ciarapica FE : Digital Twin Reference Model Development to Prevent Operators’ Risk in Process Plants. *Sustainability.* 2020 Feb;12(3):1088. 10.3390/su12031088

[42] Mikhaylov AY : Development of Friedrich von Hayekʼs theory of private money and economic implications for digital currencies. *Terra Econ.* 2021 Mar 25;19(1):53–62. 10.18522/2073-6606-2021-19-1-53-62 Reference Source

