# Supplementary material for: Smart city perspectives in post-pandemic governance: Externalities reduction policy
Source: F1000Res. 2022 Sep 12;11:1032. [Version 1] doi: 10.12688/f1000research.123195.1 (PMC9593024; doi:10.12688/f1000research.123195.1)
Supplement: Peer review history

# Peer review: Reviewer response for version 1

**DOI:** 10.5256/f1000research.135279.r153718

**Published:** 2022-10-24

**Authors:** Elena Schislyaeva

**Affiliations:** 1 Department of Innovation Economics Relations, Peter the Great St. Petersburg Polytechnic University, St. Petersburg, Russian Federation

The authors of the article rightly believe that mathematical models designed to predict the dynamics of investment projects with risky returns are necessary, first of all, for socially responsible activities. Long-term planning distinguishes the work of institutional investors such as pension funds. These financial players differ from other market participants by their incomparably longer business cycle. Accordingly, the social significance of their activities and the impact on the quality of life of all segments of the population increase. The need to develop a methodology for assessing sustainability is also dictated by the increased role of digital technologies, as well as the ability to receive the most relevant information for analysis online. In addition, as socially oriented subjects, in addition to the tasks of making a profit, the most important priority is the sustainability of the development of society and maintaining the quality of life. Such institutional investors in the world have a significant impact on the business climate due to the scale of their activities. In this work, the problem of sustainability of investment activity is investigated. The reviewed work outlines the methods and results of a study aimed at the digital transformation of methods for forecasting long-term institutional investments.

Is the rationale for developing the new method (or application) clearly explained?

Yes

Is the description of the method technically sound?

Yes

Are the conclusions about the method and its performance adequately supported by the findings presented in the article?

Yes

If any results are presented, are all the source data underlying the results available to ensure full reproducibility?

Yes

Are sufficient details provided to allow replication of the method development and its use by others?

Yes

Reviewer Expertise:

Digital logistics and Smart supply chains

I confirm that I have read this submission and believe that I have an appropriate level of expertise to confirm that it is of an acceptable scientific standard.

# Peer review: Reviewer response for version 1

**DOI:** 10.5256/f1000research.135279.r150171

**Published:** 2022-10-06

**Authors:** Bilal Khalid

**Affiliations:** 1 KMITL Business School, King Mongkut's Institute of Technology Ladkrabang, Bangkok, Thailand

**Abstract**

- The overview of the abstract was well presented, but there is still room for improvement.
- Methods: The methods should be improved to capture the type of study, procedure, population, and method of data analysis.
- The abstract will also benefit from the inclusion of study limitations and recommendations if any.
- The authors can also add one more keyword to capture the essence of the study.

**Introduction**

- The importance of the research was stated, and I commend the authors on the novel approach to presenting the study.
- The introduction makes all the arguments for the benefit of smart cities and the possible impacts of the COVID-19 pandemic. The authors have cited adequate literature to support their arguments.
- The authors were able to present the motivations for the study and highlight the importance of smart city projects in mitigating pandemics.

**Methods**

- The introductory part of the Methods section is scanty. The authors should discuss the relevant theories, models, and processes adequately to show an understanding of the topic of research.
- The research method adopted for the study seems adequate.
- The procedures, sampling techniques, and instruments have been adequately described. The researchers have clearly outlined these showing a clear understanding of the procedures, data collection, sampling technique and instruments, and method of data analysis adopted for the study. Authors should justify the use of the Fourier method in the methodology.

**Results**

- The appropriate analysis was used to present the results from the data collection process.
- The results were clearly presented and explained. The equations all follow a logical order making it an interesting read. This is the part I enjoyed the most so far in my review of this paper.
- The results section does not need further improvement. The results are clearly and lucidly presented.

**Discussion**

- The authors can improve the discussion by presenting their impacts using the equations in sequence. Show the implication of the findings based on the presented mathematical equation.
- The discussion can be improved by adding theoretical and managerial implications.
- I suggest adding at least one more paragraph to the discussion. It is too short in its current form.

**Conclusion**

- The conclusion section addresses all the points by summarizing the outcome. The authors can improve the conclusion by including the study recommendations and limitations.

**Title**

- The title is appropriate and expansive enough to support the ideas that the manuscript represents.
- The title is fine as it is.
- The title needs no further improvement.

My summary of the paper is that the topic is topical, and readers would be interested in the topic. The contributions of the paper are clear even though the discussion section does require minor improvements. The discussion should be based on the research results and how they interact with the literature for one to be able to understand the contribution of this manuscript. The findings are novel to a lesser extent. This may improve when the authors situate their discussion in the findings of the mathematical equations. The paper is not plagiarized. The paper is suitable for an international audience as it can be applied in any region. There were no country-specific concepts explained. There are minor corrections needed in the manuscript, especially in the abstract, methodology, and discussion. My overall recommendation would be to accept the article with minor corrections.

Is the rationale for developing the new method (or application) clearly explained?

Yes

Is the description of the method technically sound?

Yes

Are the conclusions about the method and its performance adequately supported by the findings presented in the article?

Yes

If any results are presented, are all the source data underlying the results available to ensure full reproducibility?

Yes

Are sufficient details provided to allow replication of the method development and its use by others?

Yes

Reviewer Expertise:

Sustainability, Business Management, Industry 4.0, Smart Cities, Technology Adoption, Consumer Behavior.

I confirm that I have read this submission and believe that I have an appropriate level of expertise to confirm that it is of an acceptable scientific standard.
